# Citizen attitudes to non-treatment decision making: a Norwegian survey

**DOI:** 10.1186/s12910-023-00900-5

**Published:** 2023-03-08

**Authors:** David Wikstøl, Morten Andreas Horn, Reidar Pedersen, Morten Magelssen

**Affiliations:** 1 Centre for Medical Ethics, Institute of Health and Society, University of Oslo, Pb. 1130, 0318 Blindern, Oslo, Norway; 2 Department of Neurology, Oslo University Hospital, Oslo, Norway; 3 MF Norwegian School of Theology, Religion and Society, Oslo, Norway

**Keywords:** Advance care planning, Attitude survey, Clinical ethics, End-of-life decision making, Minimally conscious state, Non-treatment decisions, Unresponsive wakefulness syndrome, Withdrawal of life-prolonging treatment

## Abstract

**Background:**

Decisions about appropriate treatment at the end of life are common in modern healthcare. Non-treatment decisions (NTDs), comprising both *withdrawal* and *withholding* of (potentially) life-prolonging treatment are in principle accepted in Norway. However, in practice they may give rise to significant moral problems for health professionals, patients and next of kin. Here, patient values must be considered. It is relevant to study the moral views and intuitions of the general population on NTDs and special areas of contention such as the role of next of kin in decision-making.

**Methods:**

Electronic survey to members of a nationally representative panel of Norwegian adults. Respondents were presented with vignettes describing patients with disorders of consciousness, dementia, and cancer where patient preferences varied. Respondents answered ten questions about the acceptability of non-treatment decision making and the role of next of kin.

**Results:**

We received 1035 complete responses (response rate 40.7%). A large majority, 88%, supported the right of competent patients to refuse treatment in general. When an NTD was in line with the patient’s previously expressed preferences, more respondents tended to find NTDs acceptable. More respondents would accept NTDs for themselves than for the vignette patients. In a scenario with an incompetent patient, clear majorities wanted the views of next of kin to be given some but not decisive weight, and more weight if concordant with the patient’s wishes. There were, however, large variations in the respondents’ views.

**Conclusion:**

This survey of a representative sample of the Norwegian adult population indicates that attitudes to NTDs are often in line with national laws and guidelines. However, the high variance among the respondents and relatively large weight given to next of kin’s views, indicate a need for appropriate dialogue among all stakeholders to prevent conflicts and extra burdens. Furthermore, the emphasis given to previously expressed opinions indicates that advance care planning may increase the legitimacy of NTDs and prevent challenging decision-making processes.

**Supplementary Information:**

The online version contains supplementary material available at 10.1186/s12910-023-00900-5.

## Background

Decisions about appropriate treatment at the end of life are common in modern healthcare. A central concept is *non-treatment decisions* (*NTDs*), which comprises both *withdrawal* and *withholding* of (potentially) life-prolonging treatment [1]. The EURELD study showed that NTDs were common in the six European countries studied, although varying in incidence from 6% of deaths in Italy to 41% in Switzerland [2]. NTDs are in principle clinically, ethically and legally uncontroversial in Norway. However, in practice they may give rise to significant moral problems for health professionals, patients and next of kin. Here, patient preferences and values must be taken into account. It is relevant to study the moral views and intuitions of the general population on NTDs and special areas of contention. Familiarity with the patient’s and next of kin’s preferences, attitudes and knowledge is helpful for physicians in particular. This might enable more tailored decision-making and information processes. In this context, knowledge about the population’s attitudes and variations in such attitudes could be useful [2].

### Law and guidelines on non-treatment decisions

Norwegian law, and codes of ethics such as The Norwegian Medical Association’s Code of Ethics, make clear that an NTD is different from assisted dying, which is prohibited in Norway [3–5]. Although an NTD might impact the timing of death and in this sense cause death, in NTDs, as opposed to in assisted dying, the illness is allowed to take its course and thus the illness is the (primary) cause of death. In Norwegian guidelines, withdrawal of tube feeding is included in the NTD concept. (However, limiting a patient’s oral intake or feeding would *not* be considered an NTD and would typically be regarded as unethical.) According to Norwegian law and guidelines, a patient cannot demand treatment which the responsible physician deems futile, professionally unsound or not medically indicated [4]. The latter assessment is up to the responsible physician to make. Both medical and ethical features of each particular case must be considered, and it would be difficult to prescribe detailed criteria. However, in the official Norwegian guidelines five circumstances where NTDs ought to be *considered* (although not necessarily *made*) are mentioned specifically: (1) When requested by the patient; (2) treatment is prolonging distress in the dying process; (3) treatment is prolonging a life in a state of great suffering; (4) permanent cessation of higher mental functions; and (5) (irreversible) coma [4].

The weighing of salient considerations can be challenging for the physician [1]. This is especially so when involved parties—patient, next of kin, health professionals—have different views on what is right to do. A not uncommon situation is where the patient lacks competence to consent and conflicts arise between the views of the physician(s) and those of next of kin. However, in Norwegian law the main responsibility and decision-making authority lies with the responsible physician, and not with next of kin [4]. When the patient lacks competence to consent, life prolonging treatment can only be provided when it is deemed by the responsible physician to be in the best interest of the patient and likely that the patient would have consented if competent. In such a situation, next of kin are not entitled to consent on behalf of the patient. Instead, next of kin should be asked what they know about what the *patient* would have wanted. Thus, next of kin’s *own* preferences are not given any weight in the law. There is, however, research indicating that physicians, due to pressure from next of kin, sometimes prolong treatment even though it is considered not to be in the patient’s best interest and sometimes harmful [6]. This is problematic since it might violate the principles of non-maleficence, fair allocation of resources and give next of kin a role in the decision-making that is not mandated by law. Many countries have specific laws or regulations for substituted decision making or durable power of attorney, yet this is not the case in Norway. Neither are advance directives legally binding in Norway.

Ethical problems related to end-of-life decision making are among the largest categories of ethical problems discussed in the Norwegian hospital clinical ethics committees [7, 8]. A special case often causing problems is when the patient is deemed incompetent to consent and prior preferences for treatment are unknown.

### European citizens’ attitudes to non-treatment decisions

From the last two decades, there are many studies that have charted attitudes towards assisted dying among the European public. There are also many studies of physicians’ end-of-life decision-making and attitudes towards NTDs. However, there are fewer studies of public attitudes to NTDs. Furthermore, comparison of study results is challenging because studies typically use specific case vignettes and questions rather than standardized questions/questionnaires.

In a Swedish study of both physicians and members of the general public, majorities of each group were in acceptance of withdrawing life-sustaining treatment from a hopelessly ill patient described in a case vignette [9]. In an English study of citizens, respondents were faced with a scenario involving dementia. NTDs were favoured more often when the dementia was described as more severe. In severe dementia, less than 40% would want to be resuscitated after a heart attack and 73% would want to be allowed to die. Respondents were more often in favour of life-sustaining treatment for their partner than for themselves [10]. However, another European study found that patients that had experienced ICU treatment would want active treatment far more often than physicians and nurses would if terminally ill or in a permanent disorder of consciousness [11]. In a recent study involving citizens in two Italian regions, 89% were accepting of an informed patient’s right to refuse artificial nutrition and hydration [12]. In a Dutch study, more than half of respondents would refuse life-prolonging treatment for themselves in case of advanced cancer or advanced dementia [13]. Several studies, however, show that respondents are sensitive to how scenarios and decisions are described, and that there is significant variation in attitudes with a minority apparently not being accepting of NTDs [13–17].

### Non-treatment decisions in disorders of consciousness

Nationally and internationally, the withdrawal of life-prolonging treatment from patients with severe, chronic disorders of consciousness (the unresponsive wakefulness syndrome, UWS, previously termed “persistent vegetative state”; and the minimally conscious state, MCS) has given rise to legal and ethical controversy [18]. One example is the case of Vincent Lambert, who was diagnosed with UWS in 2008 secondary to traumatic brain injury. He was kept artificially alive for years, contrary to the physicians’ views, due to court proceedings initiated by his parents. Lambert died in 2019 [19].

In a study of European health professionals, 66% agreed to withdraw treatment in UWS and 82% would not wish to be kept alive themselves. For MCS the corresponding numbers were 28% and 67% [20].

In a small 2014 survey of US residents, 40% supported the permissibility of withdrawal of treatment from patients in a vegetative state, while 18% did not [21]. Respondents were considerably less often supportive of NTD in the case of minimally conscious state (20% supported treatment withdrawal, 41% did not). In an online survey with lay participants from 32 countries, 46–49% accepted treatment withdrawal in UWS [22]. Participants more often accepted treatment withdrawal when the hypothetical case concerned themselves than someone else. In both these studies, religiously affiliated respondents were less accepting of NTDs [21, 22].

### Aim and hypotheses

With the study we aimed to understand the Norwegian public’s moral views, attitudes and intuitions concerning NTDs, with special emphasis on decision-making in disorders of consciousness and the role of next of kin. We formulated eight hypotheses, presented in Table 1, about the public’s views and attitudes.

**Table 1:** Eight hypotheses about the Norwegian public’s moral views and attitudes towards non-treatment decisions

	Hypothesis
**H1**	A majority think that withdrawing/withholding of life-prolonging treatment for UWS and MCS is morally acceptable
**H2**	There is lower acceptance of non-treatment decisions with increasing levels of patient awareness and function
**H3**	Respondents are more accepting of non-treatment decisions for themselves than for a third person
**H4**	In their evaluation of the morality of non-treatment decisions, respondents give weight to the patient’s own preferences
**H5**	In their evaluation of the morality of non-treatment decisions, a majority of respondents give weight to the costs of care and treatment
**H6**	Religious affiliation correlates negatively with acceptance of non-treatment decisions
**H7**	A large majority supports a competent patient’s right to refuse life-prolonging treatment
**H8**	A majority think that the views/decisions of health professionals should prevail over the views of next of kin when these conflict

## Methods

In December 2019, an electronic questionnaire was distributed by the commercial firm Kantar to members of their nationally representative panel of Norwegian adults. Panel members were given information about the study, which they were told would assess attitudes towards ethical issues in healthcare. 2540 panel members were invited, and 1076 responded. 1035 complete responses were received (response rate 40.7%). Responses were weighted according to gender, age, and geographical region to closer approximate the national averages. Analyses were performed on weighted data. For the demographic characteristics of respondents, see Table 2.

**Table 2:** Demographic characteristics of participants. N = 1035

	Unweighted (N (%))	Weighted (N (%))
**Age**		
Mean Under 30 30–44 45–59 60+	53.5 years 125 (12.1) 188 (18.2) 291 (28.1) 431 (41.6)	47.8 years 210 (20.3) 266 (25.7) 266 (25.7) 293 (28.3)
**Female gender**	534 (51.6)	514 (49.6)
**Highest completed education**		
Primary school Upper secondary school Higher ed./vocational school ≤ 4 years Higher education > 4 years	48 (4.6) 269 (26) 464 (44.9) 254 (24.5)	39 (3.8) 296 (28.6) 445 (43) 255 (24.7)
**Religious affiliation**		
Christian Muslim Other religion Non-religious Unanswered	533 (51.5) 6 (0.6) 13 (1.3) 420 (40.6) 63 (6)	485 (46.8) 9 (0.9) 16 (1.5) 462 (44.7) 63 (6.1)

The relevant section of the questionnaire was designed to test the eight hypotheses on attitudes to NTDs (Table 1). Other sections contained questions on priority setting [23] and advance care planning [24]. The questionnaire was presented in Norwegian. A translated version is available in Appendix 1 (see Additional file 1).

The section on NTDs consisted of ten questions. Respondents were presented with three patient vignettes. The first concerned serious disorders of consciousness; the second serious dementia in an elderly patient; and the third a patient with incurable cancer.

For the first vignette, the respondents were randomized to receive one out of two different versions, either one presenting a patient in unresponsive wakefulness syndrome (UWS, vignette 1 A) or one presenting a patient in minimally conscious state (MCS, vignette 1B). These vignettes were inspired by the vignettes used by Kondziella et al. [22], and were intended to be similar to each other in all respects except the description of the patient’s clinical state, where UWS implies that consciousness is obliterated and MCS that consciousness is minimally present.

Thus, respondents who received vignette 1 A were presented with the following: “Some patients with severe brain injury enter a state called “unresponsive wakefulness.” They alternate between sleeping and lying awake with open eyes but show no signs of consciousness or willed actions. After one year, the probability of improvement is very low. Patient M. is a 50-year-old woman with severe brain injury after a car accident three years ago. She is now in a state of unresponsive wakefulness and lives in a nursing home. During the day, the nurses put her in a chair. Her eyes are open, but she does not look at the nurses or visitors. She does not say anything and makes no intelligible sounds. When she is touched or talked to, she does not respond. She has a feeding tube connected directly to her stomach, and she has no control over urination or defecation.”

Vignette 1B read as follows: “Some patients with severe brain injury enter a so-called “minimally conscious state”. They are awake but have greatly reduced consciousness. They may show behavior that indicates a certain degree of awareness of themselves and their surroundings. After one year, the probability of improvement is low. Patient M. is a 50-year-old woman with severe brain injury after a car accident three years ago. She is now in a minimally conscious state and lives in a nursing home. During the day, the nurses put her in a chair. Her eyes are open, and sometimes she looks at the nurses or visitors and can follow objects with her eyes. She can sometimes say yes and no in response to simple situations but has no language beyond this. She is not able to move around on her own. She has a feeding tube connected directly to her stomach, and she has no control over urination or defecation.”

Each respondent was also randomized to receive one out of three additional pieces of information about the patient’s preferences. The first group were told that before the injury the patient had stated that she would *not* want to be kept alive if in need of care in a nursing home. The second group were told that the patient had previously expressed a clear preference for “full treatment”. The third group were told that patient preferences were unknown. Thus, the combination of the two vignettes and the three different varieties of patient preference yielded six different groups in total.

All respondents were then presented with the same questions (Q1-4). Three questions concerned the moral acceptability of NTDs, and the final question concerned the moral significance of the costs of care. Respondents were asked to state whether they agreed or disagreed with the following propositions: Q1. “It is here acceptable to stop the provision of fluids and nutrition. The patient will then die”; Q2. “If I was in a situation like this patient myself, I would have wanted the provision of fluid and nutrition to stop, so that I would die”; Q3. “If the patient develops pneumonia or any other serious infection, it is acceptable to refrain from treating the infection. The patient may then die”. Thus, while Q1-3 all referred to non-treatment decisions, Q1-2 concerned *withdrawing* treatment, whereas Q3 concerned *withholding* treatment. Respondents were then told that “care and treatment for this patient costs approximately 1 million NOK [≈ € 100,000] per year.” Q4 then read: “The costs of care and treatment count in favour of stopping the supply of fluid and nutrition.”

Vignette 2 then described a patient with serious dementia, reduced function and apparent suffering. The vignette read, “Patient N. is an 80-year-old man with dementia (Alzheimer’s disease). He is now bedridden much of the day but can walk with support. He lives in a nursing home. The disease has reduced his ability to think and speak, and staff now believe that he is no longer able to make his own decisions. He often groans and seems sad. In the last month, the patient has rejected all attempts at feeding. After this, he has received fluid and nutrition through a feeding tube connected directly to the stomach. The doctor and nurses are now considering whether this treatment should be continued.”

In the same way as for the preceding vignettes, respondents were randomized to receive additional information concerning the patient’s earlier expressed preferences. Respondents were asked the same three initial questions about the morality of NTDs as in the preceding vignettes. Thus, questions Q5-7 correspond to questions Q1-3. They were then presented with the following (Q8): “Imagine that the doctor and nurses think that treatment ought to be stopped. They discuss this with the patient’s closest next of kin. The next of kin wants the treatment to continue, so that the patient will live on.” They were then asked to answer the following question: “How much weight should the next of kin’s own view be accorded here?”

Finally, vignette 3 described a patient with terminal cancer who preferred not to undergo a further round of chemotherapy. The two subsequent questions concerned the right to refuse treatment. The vignette read, “Patient L. is a 70-year-old woman with incurable cancer. She has been through two series of chemotherapy to slow the progression of disease and attenuate symptoms. A third type of chemotherapy is now an option. The patient does not want this, because she had bothersome side effects of the previous drugs. However, the doctor and relatives think it is right to give it a try.” Respondents were then asked to take a stand on two questions. Q9 read, “The patient’s “no” to chemotherapy ought to be respected”. Q10 read, “In general, patients who are able to make their own choices should have the right to say “no” to all medical treatment”.

To all questions posed as propositions except Q8, the alternatives were “fully disagree”, “somewhat disagree”, “neither agree nor disagree”, “somewhat agree”, “fully agree” and “do not wish to state”.

Religiosity was determined by the answer to the question, “Which of the alternatives below best describes your worldview?” (cf. Table 2).

### Questionnaire development and statistical analyses

The questionnaire was designed to test the hypotheses and was developed in collaboration between the authors. Several physicians and experts in medical ethics gave feedback on draft versions. The electronic questionnaire was pilot tested.

Statistical analyses were performed in IBM SPSS Statistics 27. Likert scores were calculated on a 5-point scale with “fully disagree” =1 and “fully agree” =5, except for Q8 where a 4-point scale was used. Differences between groups of respondents were analysed by the Wilcoxon–Mann–Whitney test and statistical significance defined as p < 0.05.

## Results

When a non-treatment decision is in line with the patient’s previously expressed preferences, a majority of respondents support the acceptability of the NTD both in the vignette with UWS, with MCS and with dementia (Q1, Q3, Q5&Q7, Table 3). On Q1/Q5, when “fully agree” and “somewhat agree” are added together, support ranges from 66% in the UWS vignette and 64% in MCS to 71% in the dementia vignette. Respectively, 16%, 22% and 17% disagree fully or somewhat. Thus, hypothesis H1 (Table 1) was confirmed.

**Table 3:** The respondents’ answers on Q1-7 and Q9-10. Mean Likert score [1–5]. UWS = unresponsive wakefulness syndrome. MCS = minimally conscious state

Question	Case	Patient did not want treatment	Patient preference unknown	Patient wanted treatment
Q1/Q5: Acceptance of withdrawal of treatment	UWS**	3.84	3.32	3.14
	MCS	3.67	2.61	2.13
	Dementia	3.91	3.43	2.62
Q3/Q7: Acceptance of withholding of treatment	UWS*	3.60	3.53	3.39
	MCS	3.71	3.21	2.95
	Dementia	3.87	3.54	2.96
Q4: Costs favour withdrawal of treatment	UWS**	2.76	2.98	2.90
	MCS	2.73	2.44	2.22
Q2/Q6: Acceptance of withdrawal of treatment if oneself was concerned	UWS*		4.03	
	MCS		3.86	
	Dementia		3.88	
Q9: Patient’s refusal of chemotherapy should be respected	Cancer		4.57	
Q10: Competent patients’ treatment refusal should be respected	Cancer		4.52	

Differences between UWS and MCS scenarios were tested for statistical significance with the Wilcoxon–Mann–Whitney test. *p < 0.05; **p < 0.001

Support for NTD is considerably lower when patient preferences are unknown, and lower still when the patient is thought to have preferred treatment. This confirmed hypothesis H4.

When the UWS and MCS scenarios are compared, fewer support NTDs in MCS than in UWS, thus confirming H2 and the contention that NTD is deemed less acceptable with increasing levels of patient awareness and function. However, support for NTD in the dementia case is even higher.

Acceptance of withholding of treatment (Q3&7) is higher than withdrawal of treatment (Q1&5) when patient preferences are either unknown or are in favour of treatment.

Respondents are slightly more accepting of NTDs for themselves than for the patients described in the vignettes (Q2&6, Table 3). This confirmed H3.

When asked whether costs of care would count in favour of treatment withdrawal (Q4), more respondents disagreed than agreed. Thus, H5 was disconfirmed.

In general, respondents who stated a religious affiliation were less likely to accept non-treatment decisions. The effect was moderate. For instance, on Q1 (all vignette versions pooled), among religious respondents 43% supported treatment withdrawal whereas 37% did not; the corresponding numbers among non-religious respondents were 51% and 32% (p = 0.012). On Q6, which asks what respondents would have wanted for themselves in a situation like the dementia case, fewer religious than non-religious respondents would want treatment withdrawal (61% vs. 72%; p = 0.036). Thus, H6 was confirmed.

In the cancer scenario, large majorities agreed fully or somewhat that the patient’s refusal of chemotherapy ought to be respected and that competent patients’ refusals of treatment ought to be respected in general (88% in both Q9 and Q10; Table 3). On the latter question, only 1.3% disagreed fully and 3.0% disagreed somewhat. H7 was confirmed.

In the vignette involving dementia, the next of kin were portrayed as having a clear preference for treatment to continue. Some respondents (13–14%) wanted next of kin’s preference to receive decisive weight, but a majority did not, thus confirming H8 (Table 4). However, clear majorities thought that next of kin’s preferences should be given at least *some* weight. This was so even in the case where the patient’s previously expressed preference was against treatment. Although most favoured that next of kin’s views were given “some” weight, a sizeable minority would give “large” weight to these.

**Table 4:** Respondents’ views on how much weight should be accorded to the next of kin’s own view in decision-making for a patient with dementia (Q8), grouped by patient preference. N (%)

Preference	Weight						
	No	Some	Large	Decisive	Unanswered	Mean likert (1–4)	N
Patient did not want treatment	22 (6.5%)	167 (50%)	88 (26%)	45 (13%)	15 (4.5%)	2.48*	337
Patient preference unknown	14 (4.3%)	133 (41%)	118 (36%)	46 (14%)	17 (5.2%)	2.63	328
Patient wanted treatment	19 (5.1%)	130 (35%)	166 (45%)	47 (13%)	10 (2.7%)	2.67	372

*Difference between first and third group (patient does not want treatment vs. wants treatment) statistically significant, p = 0.027.

## Discussion

This survey of a representative sample of the Norwegian adult population indicates that attitudes to NTDs are often in line with national laws and guidelines. Most respondents accept NTDs, also in the context of disorders of consciousness and dementia. While many think the views of next of kin ought to be given weight, few think they should be decisive when conflicting with the views of physicians in charge of care.

Below we summarize the main tendencies in the responses. However, one of the most striking findings is the large variation in the answers, as also found in other studies [13–17]. We will return to this finding in the discussion of implications for practice.

### Acceptance of non-treatment decisions

Results indicate large acceptance of non-treatment decision making as such, including where those decisions might ultimately lead to the death of the patient. This is in line with most other European studies [9, 10, 12, 13]. Acceptance rates were higher than in the US study referenced [21]. Although NTDs will continue to give rise to conflicts and controversy in concrete situations in clinical practice, they are accepted by nearly all in general. Large support for a competent patient’s right to refuse life-prolonging treatment indicates that high value is placed on respect for individual autonomy. This is in line with findings from the World Values Survey; here, Norwegians score exceptionally high on so-called «self-expression values» (as opposed to «survival values») which include placing a high value on individual autonomy [25]. That religious affiliation was associated with lower acceptance of NTDs is in line with previous research [21, 22].

Information about the patient’s prior preferences seems to influence respondents’ views about what ought to be decided. However, we also see many respondents indicating that treatment ought to be continued in spite of previously expressed preferences to the contrary, and vice versa. Prior preferences appear to be treated simply as one of several morally relevant factors; thus, other factors, such as the clinical description of function and symptoms, might also be given significant weight.

### Making decisions for oneself versus for others

Importantly, respondents are more positive towards NTDs when speaking for themselves, than with regards to what should happen to the persons described in the vignettes. This echoes findings in several other European studies [10, 20]. One interpretation might be that when responding on behalf of oneself, the *true sentiment towards NTDs as such* comes forth. The respondents thereby show their own view on the acceptability of NTDs leading to death in cases of impaired decisional capacity.

How, then, should the lower rates of acceptance towards NTDs for the patients portrayed in the vignettes be interpreted? We suggest that when respondents are taking a stance with regards to the vignettes, they are placed in the position of making decisions impacting the life or death of another human being. This position is foreign to most ordinary people, whereas physicians might experience it regularly. Although respondents are only asked to provide their opinion on a hypothetical case, the wording of the vignettes demands that the respondent makes a decision, following which the hypothetical patient will die.

It is well known that even physicians might experience end-of-life decision making as a burden, despite their training and experience with such decisions [26]. It is only natural that ordinary people may be shrinking back from shouldering that responsibility, even when only taking place in theory. Respondents may be naturally cautious about making decisions with devastating consequences on behalf of other people, particularly when faced with the scant information provided in the vignettes. On the other hand, when making a decision with regards to oneself it might be easier to accept that one has received sufficient information to make up one’s own mind. In addition, respondents might think that there is some uncertainty concerning the patient’s prior expressed wishes and their applicability to the decision at hand. This could call for reluctance in accepting NTDs.

Responses indicate that respondents are more reluctant towards withdrawing treatment, rather than withholding treatment, in the scenario where the patient wants treatment. (A caveat, however, is that the relevant questions involve different treatment modalities, so they are not fully equivalent.) This is a well-known phenomenon; even though withholding and withdrawing treatment are usually seen as ethically equivalent, they may be emotionally different [27]. The prospect of actively removing treatment from a patient who previously indicated that he or she wanted treatment, is understandably challenging. Again, this need not necessarily be seen as an expression of the view that patient preferences should override medical decisions. Rather, it might be an expression of the natural tendency of respondents to be cautious about making decisions on behalf of others.

### Next of kin’s role in non-treatment decision-making

As discussed above, Norwegian health law gives next of kin a limited and clearly demarcated responsibility when the patient lacks competence to consent. The majority of our respondents do not challenge the physician’s decision-making authority. However, many appear to want next of kin’s own preferences (as distinguished from next of kin’s report of the *patient’s* prior expressed preferences) to be given some weight in decision-making, even when the next of kin’s preferences *run contrary to* the patient’s previously expressed preferences. This would go against present health law but might be more in line with actual practice [28]. More research is needed to characterise the role that next of kin and their preferences have in actual, clinical decision-making in this context.

### Some implications for practice

The findings might carry consequences for how physicians interact with next of kin of patients lacking decisional capacity for whom NTDs are considered. The large variations in the responses indicate a strong need for careful dialogue with patients and next of kin about this kind of questions. To be able to clarify and adapt to individual preferences, but also to clarify and comply with the rules, and possibly sometimes to make informed and well-justified exceptions, is important. Furthermore, the emphasis given to previously expressed wishes and next of kin’s views points to the need for appropriate advance care planning.

Furthermore, what might at first glance appear as a conflict of opinion—the physician wants to stop treatment, the relative wants it to continue—might actually mean that the relative is naturally cautious about being the one to agree with and support the physician in the decision to let a loved one die. This is probably more so if previous dialogue and advance care planning has been sparse or non-existent. Both in advance care planning and shared decision making “here and now”, information about the legal roles and rights should be given. In Norway, the physicians are the ones responsible for making the decisions. Thus, the relatives may “lower their shoulders” and be more forthright with their true feelings about the matter, and also give more information about their knowledge about what the patient would have wanted.

Another application of these results could be the active use of advance care planning to avoid overtreatment [24]. Given the responses regarding what one would want for oneself, it seems that a clear majority would not want to be kept artificially alive, in the case of a serious disorder of consciousness. Through advance care planning such values and preferences might be expressed and recorded in patient charts. This would then constitute important information which would likely be helpful in clinical decision-making about treatment when the patient has lost competence.

A particular point of interest is the rather sizeable minority that *do* want to be kept alive, despite being in a state of UWS, MCS or dementia. Obviously, health care professionals need to be aware that this subgroup exists. One cannot automatically assume that “no patient would want this life”. Physicians therefore need to engage with patients and next of kin with an open mind, treading carefully, while trying to elucidate the attitudes of the patient or family in question.

Still, according to conventional medical practice and ethics in Norway, keeping alive patients in an UWS or even MCS with little hope of improvement is challenging as well [4]. Whether continued treatment constitutes beneficence is questionable. The risk of doing harm in keeping patients alive in a state of profound disability contrary to the patient’s values is clear. Respecting patient autonomy is difficult when there is no reliable, valid account of the patient’s prior preferences. Allocating scarce health care resources to patients who lack awareness of their existence is arguably not in accordance with distributive justice. Respondents, however, did not indicate costs as an important argument in decision-making here.

The study indicates that although a minority view, some patients and next of kin will find non-treatment decisions ethically problematic. This is in line with findings from other European studies [9, 12]. Our view is that the best way to handle such patients and families would be to provide factually correct, evidence-based information regarding the state of the patient, the prognosis, the risks of suffering and the futility of further treatment. Frequently, information processes such as these are time-consuming, and need to be repeated. In order to achieve a fruitful environment for such conversations, it is important that physicians are aware that critical attitudes to NTDs exist, and that they are legitimate from the patient point of view, even though they go against the prevailing medical and ethical views.

### Limitations

According to the belief-sampling model of survey response, the attitudes tested by such surveys are seen as “a kind of memory structure that contains existing evaluations, vague impressions, general values, and relevant feelings and beliefs” [29]. Thus, answers based on this “memory structure”, from respondents presumed to be largely unfamiliar with these particular ethical questions or situations, are more “intuitions” than they are considered ethical judgments. The results of the survey should be interpreted in this light.

Although a non-response bias cannot be ruled out, the moderately high response rate of 40.7% lends some support to the validity of the findings. In the invitation to participate in the survey, panel members were told the survey would assess “attitudes towards ethical issues in healthcare”. Conceivably, respondents with an above average interest in the topic could have been more likely to respond.

A limitation in surveys asking respondents to take stands on “paper cases” is that certain simplifications must necessarily be made. The phrasing of the information and questions involves a risk of these being interpreted differently, or of outright misunderstandings. Respondents are expected to take a stand based on very little information and without the opportunity for follow-up questions or dialogue with experts. This marks an important contrast with real-life decision making.

## Conclusion

This survey of a representative sample of the Norwegian adult population indicates that attitudes to NTDs are often in line with national laws and guidelines. Many respondents accept NTDs, also in the context of disorders of consciousness or dementia. However, there are large variations in the responses. A majority want next of kin’s own views to be accorded some weight. These responses are sometimes in friction with Norwegian health laws, something which indicates that careful dialogue, shared decision making, and advance care planning should be given high priority. This may prevent conflicts, unnecessary burdens, and overtreatment.

## Supplementary Information

**Additional file 1. Appendix 1:** Questionnaire translated from the original Norwegian into English.

## Data Availability

The data are available from the corresponding author on reasonable request.

## Abbreviations

NTD: Non-treatment decision
MCS: Minimally conscious state
UWS: Unresponsive wakefulness syndrome
Q: Question
