# Supplementary material for: Citizen attitudes to non-treatment decision making: a Norwegian survey
Source: BMC Med Ethics. 2023 Mar 8;24:20. doi: 10.1186/s12910-023-00900-5 (PMC9993678; doi:10.1186/s12910-023-00900-5)
Supplement: Supplementary file 1 — Additional file 1. Appendix 1: Questionnaire translated from the original Norwegian into English. [file 12910_2023_900_MOESM1_ESM.docx]

**Citizen attitudes to non-treatment decision making: A Norwegian survey**

**Appendix 1: Questionnaire translated from the original Norwegian into English**

Which of the alternatives below best describes your worldview?

(Christian – Muslim – Other religious worldview – Atheist/agnostic – Another non-religious worldview – Do not wish to state)

Have you during the last 6 months been to church, house of worship, mosque, synagogue, etc?

(Yes, church – Yes, house of worship – Yes, mosque – Yes, synagogue – Yes, other – None of these – Do not wish to state)

How many times have you been in contact with a general practitioner in the last 12 months?

How many times have you been in contact with public hospitals in the last 12 months?

To what degree do you have trust in the following:

Your GP (0= have no trust at all, 10= have full trust, Do not wish to state)

The public health services

That you will receive correct treatment if you become a patient in hospital

That you will receive correct information if you become a patient in hospital

*Respondents randomized to one of two cases – questions are the same*

1) Some patients with severe brain damage enter a condition called "unconscious wakefulness". They alternate between sleeping and being awake with open eyes but show no signs of consciousness or voluntary actions. After a year, the probability of improvement is very low.

Patient M. is a 50-year-old woman with severe brain damage after a car accident three years ago. She is now in a state of unconscious wakefulness and lives in a nursing home. By day, the nurses put her in a chair. Her eyes are open, but she sees neither the nurses nor any visitors. She says nothing and makes no understandable sounds. When she is touched or talked to, she does not respond. She has a feeding tube connected directly to her stomach, and she has no control over urination or bowel movements.

2) Some patients with severe brain damage enter a so-called "minimal conscious state". They are awake but have greatly reduced consciousness. They may have behaviours that indicate a certain degree of experience of themselves and their surroundings. After a year, the probability of improvement is low.

Patient M. is a 50-year-old woman with severe brain damage after a car accident three years ago. She is now in a minimally conscious state and lives in a nursing home. By day, the nurses put her in a chair. Her eyes are open, and sometimes she looks at the nurses or visitors and can follow things with her eyes. She can sometimes say yes and no in response to simple situations but has no language beyond that. She is unable to move around on her own. She has a feeding tube connected directly to her stomach, and she has no control over urination or bowel movements.

*Respondents are then randomized to receive one of three additions (same for both main groups)*

I) Prior to the injury the patient expressed that she did not want to be kept alive if in need of nursing home care.

II) Prior to the injury the patient expressed that if she became dependent on nursing home care, she would nevertheless want full treatment.

III) It is not known whether the patient prior to the injury expressed any attitude to treatment if she were to become dependent on nursing home care

You will now receive some claims connected to the information you read on the previous page. How much do you agree or disagree with the following:

**Q1.** It is here acceptable to stop the provision of fluids and nutrition. The patient will then die.

(Fully disagree – Somewhat disagree – Neither agree nor disagree – Somewhat agree – Fully agree – Do not wish to state)

**Q2.** If I was in a situation like this patient myself, I would have wanted the provision of fluid and nutrition to stop, so that I would die.

(Fully disagree – Somewhat disagree – Neither agree nor disagree – Somewhat agree – Fully agree – Do not wish to state)

**Q3.** If the patient develops pneumonia or any other serious infection, it is acceptable to refrain from treating the infection. The patient may then die.

(Fully disagree – Somewhat disagree – Neither agree nor disagree – Somewhat agree – Fully agree – Do not wish to state)

**Q4.** Care and treatment for this patient costs approx. one million kroner per year. The cost of care and treatment counts in favour of stopping the supply of fluid and nutrition.

(Fully disagree – Somewhat disagree – Neither agree nor disagree – Somewhat agree – Fully agree – Do not wish to state)

*New case – to all respondents:*

Patient N. is an 80 year old man with dementia (Alzheimer's disease). He is now asleep much of the day but can walk with support. He lives in a nursing home. The disease has impaired the ability to think and talk, and the staff now believes that he is no longer able to make his own choices. Often, he whimpers and seems sad. During the past month, the patient has rejected all attempts at feeding. After that, he has received fluid and nutrition through a feeding tube connected directly to the stomach. The doctor and nurses are now considering whether this treatment should continue.

*Respondents are then randomized to receive one of three additions:*

I) Prior to the injury the patient expressed that he did not want to be kept alive if in need of nursing home care.

II) Prior to the injury the patient expressed that if he became dependent on nursing home care, she would nevertheless want full treatment.

III) It is not known whether the patient prior to the injury expressed any attitude to treatment if he were to become dependent on nursing home care

You will now receive some claims connect to the information you read on the previous page. How much do you agree or disagree with the following:

**Q5.** It is here acceptable to stop the provision of fluids and nutrition. The patient will then die.

(Fully disagree – Somewhat disagree – Neither agree nor disagree – Somewhat agree – Fully agree – Do not wish to state)

**Q6.** If I was in a situation like this patient myself, I would have wanted the provision of fluid and nutrition to stop, so that I would die.

(Fully disagree – Somewhat disagree – Neither agree nor disagree – Somewhat agree – Fully agree – Do not wish to state)

**Q7.** If the patient develops pneumonia or any other serious infection, it is acceptable to refrain from treating the infection. The patient may then die.

(Fully disagree – Somewhat disagree – Neither agree nor disagree – Somewhat agree – Fully agree – Do not wish to state)

**Q8.** Imagine that the doctor and nurses believe that treatment should be stopped. They discuss this with the patient's closest next of kin. The next of kin wants the treatment to continue, so that the patient will live on. How much weight should the relatives' own view be accorded here?

(No weight – Some weight – Large weight – Decisive weight, next of kin should decide – Do not wish to state)

Patient L. is a 70 year old woman with incurable cancer. She has been through two series of chemotherapy to slow the progression of disease and attenuate symptoms. A third type of chemotherapy is now an option. The patient does not want this, because she had bothersome side effects of the previous drugs. But the doctor and relatives think it is right to make a trial. How much do you agree or disagree with the following:

**Q9.** The patient’s “no” to the chemotherapy should be respected.

(Fully disagree – Somewhat disagree – Neither agree nor disagree – Somewhat agree – Fully agree – Do not wish to state)

**Q10.** In general, patients who can make their own choices should have the right to say “no” to all medical treatment.

(Fully disagree – Somewhat disagree – Neither agree nor disagree – Somewhat agree – Fully agree – Do not wish to state)
